# Dynamic thiol/disulfide homeostasis as oxidative stress marker in diabetic ketoacidosis

**DOI:** 10.3906/sag-1904-55

**Published:** 2021-04-30

**Authors:** Yavuz OTAL, Fatih Ahmet KAHRAMAN, Güllü F. HAYDAR, Özcan EREL

**Affiliations:** 1 Department of Emergency Medicine, Ankara City Hospital, Ankara Turkey; 2 Department of Emergency Medicine, Faculty of Medicine, Yıldırım Beyazıt University, Ankara Turkey; 3 Department of General Surgery, Ankara City Hospital, Ankara, Turkey Turkey; 4 Department of Medical Biochemistry, Faculty of Medicine, Yıldırım Beyazıt University, Ankara Turkey

**Keywords:** Diabetic ketoacidosis, thiol/disulfide homeostasis, oxidative stress

## Abstract

**Background/aim:**

The aim of present study was to investigate the dynamic thiol/disulfide homeostasis as oxidative stress marker in diabetic ketoacidosis (DKA).

**Materials and methods:**

A total of 77 participants consisting of 32 patients with DKA and 45 healthy volunteers were included in the study. Thiol/disulfide homeostasis (TDH) [total thiol-native thiol/disulfide changes] were measured in both groups (patient group and control group) using a brand new method developed by Erel and Neselioglu. Half of the difference between total thiol and native thiol concentrations gives the amount of disulfide bond.

**Results:**

Total thiol, native thiol, and disulfide levels in blood were found to be low. The levels of total thiol (P < 0.001) and native thiol (P < 0.001 ) were significantly lower in patients with DKA than in the control group. At the same time, the level of disulfide was nonsignificantly lower in the patient group than the control group (P = 0.388). The level of IMA was higher in the patient group than in the control group (P < 0.001).

**Conclusion:**

The total thiol, native thiol, and disulfide levels in DKA decrease in favor of oxidative stress.

## 1. Introduction

Diabetic ketoacidosis (DKA) is characterized by the presence of ketone bodies in the blood and metabolic acidosis in conditions of hyperglycemia or euglycemia. Hyperglycemia is one of the most important risk factors for the development of vascular complications. Hyperglycemia aggravates the clinical situation by leading to oxidative stress. In addition, the elevation of ketone bodies in the blood also plays a role in the increase of the oxidative stress. It was shown that the oxidative stress is a contributing factor to the onset and progression of the diabetes and its associated complications [1]. In an oxidative environment, some results that can lead to diabetes mellitus are development of insulin resistance, beta cell dysfunction, impaired glucose tolerance, and mitochondrial dysfunction. Oxidative stress may come to existence from a series of different sources such as disease status or lifestyle including ketosis episodes, sleep restriction, and excessive nutrient intake [1].

Thiols, most commonly known as mercaptans, are the compounds that contain a sulfhydryl group (-SH) [2]. The plasma thiol pool is mostly composed of albumin thiols and low molecular weight thiols such as cysteinylglycine, cysteine (Cys), homocysteine, glutathione, and γ-glutamylcysteine [3]. The thiol/disulfide homeostasis (TDH) has a vital importance for the organism. TDH plays a critical role in the detoxification process and it was shown that the thiols have activities in the antioxidant protection, signal transduction, enzymatic regulation, apoptosis, and cellular signaling mechanisms [4,5]. The TDH was measured unilaterally until 2014, but it is now measured bilaterally using a brand new method developed by Erel and Neselioglu [6,7]. In the present study, we investigated the role of thiol/disulfide homeostasis measured with a brand new method as an indicator of oxidative stress in etiopathogenesis of DKA patients presenting to the emergency department.

## 2. Materials and methods 

Ethics committee approval was obtained for the present study from Yıldırım Beyazıt University (28.11.2018 / 247). Informed consent was obtained from all the participants in the study. A total of 77 blood samples were collected from 32 patients and 45 healthy volunteers. The blood samples were sent to the laboratory without wasting time for the examination of biochemical values and thiol-disulfide parameters. For sample selection, the patients diagnosed with DKA were included in the study. Blood samples were taken from the patients as soon as they were diagnosed and before the treatment started. The criteria of the American Diabetes Association (ADA) were used for the definition of DKA as follows: blood glucose levels higher than 250 mg/dL, the existence of an anion gap greater than 10, bicarbonate levels lower than 18 mEq/L, and ketonemia or significant ketonuria with blood pH lower than 7.3. The patients who had a systemic disease, hypertension, cancer, renal failure, trauma; the patients who were pregnant or under 18 years of age; and the patients who died, were excluded from the study.

### 2.1. Measurement of thiol/disulfide homeostasis parameters

Thiol/disulfide homeostasis tests were measured by automated spectrophotometric method described by Erel and Neselioglu. Briefly, disulfide bonds were first reduced to form free functional thiol groups with sodium borohydride. Unused reductant sodium borohydride was consumed and removed with formaldehyde to prevent reduction of DTNB (5,5’-dithiobis-(2-nitrobenzoic) acid), and all of the thiol groups including reduced and native thiol groups were determined after the reaction with DTNB. Half of the difference between the total thiols and native thiols provides the dynamic disulfide amount. After the determination of native and total thiols, disulfide amounts, Index-1 disulfide/native thiol percent ratios ([SS]/ [SH] ×100) Index-2 : disulfide/total thiol percent ratios (SS]/ [SH+SS] ×100) and Index-3 : native thiol/total thiol percent ratios([SH]/ [SS+SH] ×100) were calculated [7] . 

### 2.2. Measurement of the IMA (ischemia- modified albumin)

Measurement of IMA levels was obtained using venous blood samples on admittance within 1 h. Specimens were stored for 30 min at room temperature and then centrifuged at 3500 rpm for 5 min. Later, the samples were transferred to Eppendorf tubes and stored at –80 °C until analysis. Albumin cobalt binding test was used to detect the presence of IMA. This test was performed by adding 50 mL 0.1% cobalt (II) chloride (CoCl2,6H2O) (Sigma-Aldrich Chemie GmbH Riedstrasse 2, Steinheim, Germany) to the patient serum. After mixing, followed by 10 min of incubation to allow for albumin cobalt binding, 50 mL of 1.5 mg/mL dithiothreitol was added. After mixing followed by 2 min of incubation, 1.0 mL of a 0.9% sodium chloride solution was added in order to reduce the binding capacity. The blank was prepared similarly with distilled water instead of dithiothreitol. The absorbance of samples was measured at 470 nm using a spectrophotometer. The results were expressed as absorbance units (ABSU).

### 2.3. Statistical analysis

The statistical analyses were performed using the IBM SPSS Statistics (Version 22) computer program (IBM, Armonk, NY, USA, 2011). Distribution of variables was evaluated by using the Kolmogorov–Smirnov/Shapiro–Wilk tests. Normally distributed data variables were compared with paired Student’s t-test or Student’s t-test. Nonnormally distributed data variables were compared using the Wilcoxon rank test or the Mann–Whitney U test. Chi-square test was used for categorical variables for two group comparisons. The correlation analyses were evaluated by Pearson’s or Spearman correlation test. A P-value of <0.05 was considered statistically significant.

## 3. Results

A total of 77 participants consisting of 32 patients with DKA and 45 healthy volunteers were included in the study. There was no statistical difference between the groups in terms of age, sex, and body mass index (Table 1). 

**Table 1:** Demographic characteristics and thiol/disulfide homeostasis values of the healthy group, DKA patient groups.

Variable	Healthy control mean ± SD (n: 45)	DKA patients mean ± SD (n: 32)	P-value
Age (years)	42.6±14.4	41.09±18.6	0.82
Sex			
Male (n/%)	51 (51.5%)	17(53%)	0.47
Female (n/%)	48 (48.5%)	15(47%)	0.47
BMI (kg/m2)	23.2 ± 4.7	25.1 ± 5.4	0.308
Glucose, mg/dL	90 ± 14.4	455.2 ± 28.5	<0.001
Urea, mg/dL	32.1 ± 9.4	55.5 ± 45.6	<0.001
Creatinine, mg/dL	0.84 ± 0.3	1.5 ± 1.4	<0.001
Na (sodyum), mEq/L	138.4 ± 3.1	142.7 ± 7.3	<0.001
K (potassium ) mEq/L	4.2 ± 0.5	4.5 ± 0.7	<0.001
GFR, mL/dk/1.73 m2	112 ± 11.6	74.4 ± 37.8	<0.001
Blood ketones, mmol/L	<0.5	2.5 ± 0.69	<0.001
Urine ketones, number of positive patients	Negative	+ (3)++ (14)+++ (6)++++ (9)	<0.001
Bicarbonate, mmol/L	24 ± 1.3	14.3 ± 5.4	<0.001
pH	7.39 ± 0.23	7.23 ± 0.15	0.036
Lactate, mmol/L	1.04 ± 0.4	3.4 ± 3.9	<0.001
pCO2	24.9 ± 1.2	28.1 ± 7.8	0.034
BE	+1.4 ± 1.1	-11.9 ± 9.4	<0.001
Native thiol [SH], µmol/L	401.8 ± 82.6	298.5 ± 70.5	<0.001
Total thiol [SH+SS], µmol/L	434.6 ± 81.3	330.9 ± 71.8	<0.001
Disulfide [SS], µmol/L	36.7 ± 15.6	16.3 ± 8.2	0.388
Index-1 [SS]/ [SH] ×100	4.5 ± 2.5	5.4 ± 2.9	0.226
Index-2 [SS]/ [SH+SS] ×100	4.2 ± 2.2	4.7 ± 2.4	0.238
Index-3 [SH]/ [SS+SH] ×100	96.6 ± 4.4	90.6 ± 4.8	0.238
IMA, ABSU	66.7 ± 9.3	74.5 ± 5.5	<0.001

**Figure 1:**
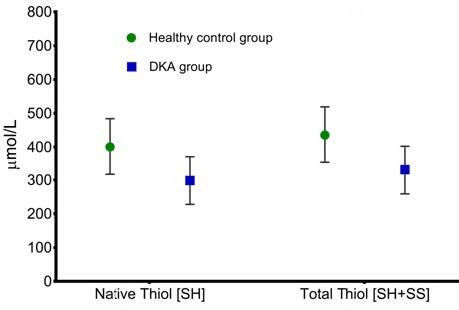
Native and total thiol levels in healthy control and DKA groups.

**Figure 2:**
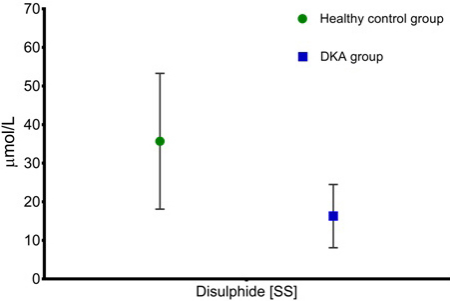
Disulfide levels in healthy control and DKA groups.

The values of the thiol/disulfide homeostasis parameters and other parameters in the patient and control groups are shown in Table 1. While it was seen that the total thiol level and the native thiol level were statistically significantly low (P *<* 0.001), it was found that the level of disulfide was low but it was not statistically significant (P *=* 0.388) (Figures 1 and 2). There was no significant change in the calculated Index-1, Index-2, and Index-3 values. 

Considering the comparison of IMA parameters in the control and DKA groups, IMA levels were high as seen in Table 1 (P < 0.001). 

The results of correlation analysis between blood glucose, urea, creatinine, and blood gas values and thiol-disulfide parameters are shown in Table 2. According to these results, there is a statistically significant negative correlation.

**Table 2:** The correlation analysis between thiol, disulfide, IMA parameters and the other parameters in DKA patients.

Native		thiol Total thiol			Disulfide			IMA		
Parameters	r	P	r	P	r	P	r		P	
Glucose	-0.318	< 0.001**	-0.328	< 0.001**	-0.068	0.442	0.200		<0.05*	
Urea	-0.232	<0.05*	-0.243	<0.05*	-0.063	0.521	0.103		0.294	
Creatinine	-0.213	< 0.05*	-0.225	<0.05*	-0.156	0.112	0.072		0.413	
Lactate	-0.455	<0.05*	-0.471	<0.05*	-0.148	0.344	0.171		0.279	
Bicarbonate	-0.384	<0.05*	-0.359	<0.05*	0.083	0.651	0.023		0.903	
pCO2	-0.380	<0.05*	-0.353	<0.05*	0.106	0.479	0.156		0.301	
Base excess	-0.419	<0.05*	-0.361	<0.05*	0.225	0.216	0.203		0.274	

*P < 0.05 and **P < 0.001 indicate the statistical significance according to the Pearson or Spearman correlation testIMA: ischemia-modified albumin

## 4. Discussion

Dynamic thiol/disulfide homeostasis plays a critical role in detoxification, antioxidant protection, apoptosis, signal transduction, regulation of enzymatic activity and transcription factors, and cellular signaling mechanisms [4,5] . Thiol protein groups are important antioxidants that constitute 52.9% of total serum antioxidant capacity in healthy individuals. Previously thiols could be measured unilaterally [6]. Erel and Neselioglu defined a new automatic test that determines the dynamic thiol/disulfide homeostasis. Serum total thiol and native thiol levels were measured by this method and disulfide concentrations were calculated with these levels [7]. Thiols are accepted as essential antioxidant buffers because they are in constant interaction with almost all physiological oxidants [8–11]. This test shows changes in the thiol or disulfide levels. It was shown that the degenerative diseases such as chronic kidney disease, cardiovascular disease, chronic inflammation, autoimmunity, and hyperglycemia were associated with oxidative stress [12,13]. 

In different studies, it was asserted that there is an association between the oxidative stress and diabetes, and that the metabolic damage increases in direct proportion to the increase in severity of oxidative stress. In addition, serum thiol levels were found to be low in the same studies [14–16]. 

Hyperglycemia is one of the most important risk factors for the development of vascular complications. It is known that hyperglycemia leads to the increased oxidative stress and the dysfunction of monocyte and endothelial cells. In patients with type 1 diabetes, ketosis (hypercetonemia) is often seen in addition to hyperglycemia. In DKA; hyperglycemia, ketonemia, and metabolic acidosis further increase oxidative stress. Elevated oxidative stress levels in ketotic patients can play a significant role in the development of vascular inflammation. This status may contribute to the increased incidence of vascular disease and complications associated with type 1 diabetes [14–16]. There is another study showing that hyperglycemia alone increases the oxidative stress and the ratio varies in favor of oxidative stress in TDH [17]. 

In many different clinical conditions, TDH was examined to show the severity of oxidative stress and significant results were found [18–23]. In our study, it was found that total thiol and native thiol levels were significantly lower in DKA. These findings show us the presence of oxidative stress in DKA, whereas the decrease in disulfide level shows us that there was advanced oxidation process. While the disulfide bonds are formed by the metabolism of thiol, the disulfide level that did not increase despite increased thiol usage in DKA makes us think that there was advanced oxidation of proteins. It is seen that the level of disulfide is low due to the transformation of protein to its end products as a result of protein advanced oxidation. There are many studies on advanced oxidation protein end-metabolism products in different diseases [24–27]. These changes formed in the thiol-disulfide balance suggest that it may be a brand new marker indicating the severity of the disease in patients with DKA. 

Ischemia-modified albumin (IMA), protein carbonyl and superoxide dismutase are other markers for oxidative stress. IMA is an altered type of serum albumin that forms under conditions of oxidative stress, and it increases due to oxidative stress after acute ischemia. IMA returns to normal levels within hours after reperfusion. IMA is a sensitive marker in myocardial infarction, peripheral vascular diseases, chronic renal disease, and diabetes mellitus [28–30]. We also evaluated the significance of IMA for DKA. We detected that the IMA has the potential to be used as a marker similar to thiol/disulfide homeostasis for showing oxidative stress.

In our study, the balance of thiol/disulfide was changed in favor of thiol, and the decreases in disulfide level were not found significant. These results are significant in terms of thiol/disulfide balance indicating oxidative stress. We think that it may play a role in etiopathogenesis of DKA.

In conclusion dynamic thiol/disulfide homeostasis in DKA changes in favor of oxidative stress. TDH may contribute to the etiopathogenesis of DKA.

There are some limitations to the present study. The study population is small. Dynamic thiol/disulfide homeostasis is affected by various diseases and conditions. In the patients with unknown etiology of diabetic ketoacidosis, there may be conditions affecting the oxidative stress. Does the giving thiol sources as treatment contribute to the reduction of oxidative stress in patients with diabetic ketoacidosis? Does it have a role in etiopathogenesis? Further studies are needed to find answers to these questions.
